# Methylation-centric epigenetic regulation in dilated cardiomyopathy: mechanisms, metabolic interplay, and translational potential

**DOI:** 10.3389/fcvm.2026.1785320

**Published:** 2026-05-11

**Authors:** Yilingrui Wang, Yang Zhang, Zun Mao, Zhiwei Xu

**Affiliations:** 1 Department of Cardiothoracic Surgery, The Affiliated Huaian No.1 People’s Hospital of Nanjing Medical University, Huai’an, Jiangsu, China; 2 Department of Anesthesiology, The Affiliated Huaian No.1 People’s Hospital of Nanjing Medical University, Huai’an, Jiangsu, China; 3 Anhui Key Laboratory of Infection and Immunity, Department of Pathology, Center for Innovation in Basic Medical Sciences, Bengbu Medical University, Bengbu, Anhui, China

**Keywords:** dilated cardiomyopathy, DNA methylation, epigenetics, histone methylation, m6A, metabolic regulation, therapeutic target

## Abstract

Dilated cardiomyopathy (DCM) is one of the main causes of heart failure and heart transplantation, with complex causes involving multiple factors such as genetics, epigenetics, and environment. In recent years, methylation, as a key epigenetic regulatory approach, has increasingly garnered attention for its role in the mechanisms of DCM onset. This review comprehensively summarizes the mechanisms, functions, and metabolic regulatory characteristics of DNA methylation, histone methylation, and RNA N6-methyladenosine (m6A) modification in the occurrence and development of DCM. Literature was selected from PubMed and Web of Science, focusing on studies related to methylation and DCM published in recent years, as well as comprehensive and well-structured review articles. Studies have shown that these three methylation modifications collectively influence the phenotypic characteristics of DCM by regulating processes such as gene expression, metabolic homeostasis, inflammatory responses, fibrosis, and cell death in cardiomyocytes. Moreover, there is cross-regulation among these modifications, which is closely related to carbon metabolism and the tricarboxylic acid cycle. Further exploration of methylation regulatory mechanisms not only helps reveal the underlying basis of DCM but also opens new directions for targeted therapy and precision medicine. This article aims to summarize existing research advancements, explore the clinical translation potential of methylation-related mechanisms, and identify key questions and challenges for future studies.

## 1 Introduction

Cardiomyopathies present four primary morphologic features: dilated, hypertrophic, restrictive, and arrhythmogenic, with the latter including both right ventricular and left ventricular subtypes. Among these, dilated cardiomyopathy (DCM) is defined by an enlarged and poorly contractile left ventricle, along with impaired ejection ability, resulting in insufficient blood flow to meet the body's metabolic demands (1). As a leading cause of heart failure, DCM is a primary indication for heart transplantation (2). Approximately 1 in 250 individuals will develop DCM during their lifetime (3), with the condition accounting for about 60% of childhood cardiomyopathies (4). Despite epidemiological limitations, DCM is implicated in up to 40% of patients enrolled in clinical trials for heart failure with reduced ejection fraction, in those requiring hospitalization for heart failure, or in candidates for cardiac transplantation (5). Regarding its etiology, DCM can arise from pathogenic gene variants, infections, autoimmunity, toxins (e.g., ethanol, recreational drugs, and cancer therapy), endocrinopathies, and tachyarrhythmias. Additionally, modifiers such as hemodynamic and hormonal changes can exacerbate cardiomyopathy without direct contribution (6). Importantly, DCM is a heterogeneous disorder with diverse etiologies, and the determinants of its clinical phenotype are highly complex. In this review, we primarily focus on non-ischemic DCM, while also incorporating insights from relevant experimental models (e.g., pressure overload and doxorubicin-induced injury) to help elucidate shared epigenetic mechanisms. In the clinical evaluation of DCM, baseline hematologic and metabolic profiles, as well as a standard electrocardiogram, are essential parameters. Additionally, ambulatory and dynamic cardiac monitoring can help assess the patient's arrhythmic burden and stratify risk. At present, standard treatment for DCM with reduced left ventricular ejection fraction (LVEF <40%) focuses on heart failure management to promote reverse remodeling, reduce ventricular dilation, and improve cardiac function (7). However, the widely used “one-size-fits-all” approach in heart failure guidelines may not fully address the complexities of DCM. Advances in understanding the genetic underpinnings of DCM will undoubtedly provide new insights into the progression of this disease. Emerging treatments include gene therapy for gene replacement or genome editing via CRISPR/Cas9 technology, signaling pathway modifiers, and other targeted strategies (8, 9). With the deepening research on epigenetic mechanisms, an increasing number of epigenetic drugs are entering clinical studies aiming to treat cardiovascular diseases such as amplified cardiomyopathy by modulating DNA methylation, histone modifications, and non-coding RNAs. For example, DNMT inhibitor 5-aza-2 -deoxycytidine (5-aza-dC) has been shown to ameliorate atherosclerosis by inhibiting macrophage inflammation (10). Epigenetic modifications can also serve as potential biomarkers for DCM to support early diagnosis, risk assessment, and treatment decisions. Glezeva et al. detected high methylation of five genes (*HEY2, MSR1, MYOM3, COX17*, and *miRNA-24-1*) in the diaphragm tissue of DCM patients in the heart failure study cohort, and low methylation of three genes (CTGF, MMP2, and *miRNA-155*) (11). Detecting these differentially methylated genes may help diagnose DCM and assess its prognosis.

The genetic basis of DCM has been extensively documented, with numerous DCM-related genes identified, such as *TTN, LMNA, PLN*, and *SCN5A*. Mutations in these genes are routinely screened as part of the clinical diagnostic process for familial DCM (1). There is an emerging recognition that phenotypic traits are not only determined by genetic variants of coding sequences at DNA level but also by the functional state of the genome. Aside from conventional Mendelian genetics, disease susceptibility is also influenced by various epigenetic modifications. Consequently, epigenetic studies mostly focus on regulatory mechanisms and functional consequences of gene expression changes rather than gene sequence alterations (12). Epigenetics refers to a regulatory mechanism that can sustain alternative gene functions, expressions, or activities without altering the DNA sequence itself. It is recognized as a primary regulatory system through which cells adapt to environmental changes. Epigenetic modifications primarily modulate the function and expression of genes associated with cardiovascular diseases, via mechanisms such as DNA methylation, histone modifications, and regulation by noncoding RNAs, thereby contributing to the progression of cardiovascular pathologies (13). Recent research has revealed significant associations between epigenetic modifications and the pathogenesis of metabolic cardiovascular disorders, such as congenital heart disease, heart failure, cardiomyopathy, hypertension, and atherosclerosis (14). In this review, we focus on the contribution of epigenetic modifications to the pathogenesis of dilated cardiomyopathy.

**Figure 1:**
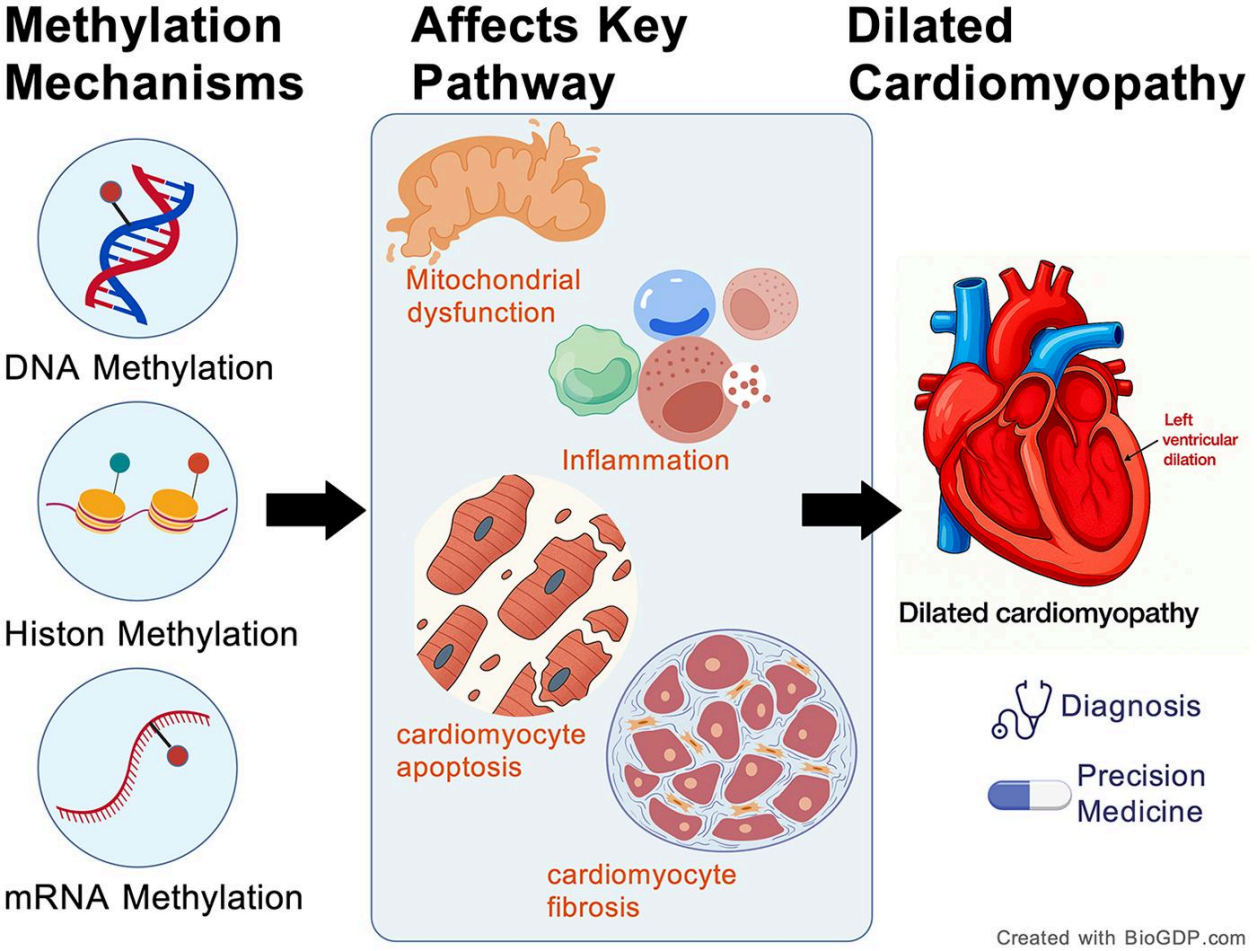
Involvement of DNA, histone and mRNA methylation in dilated cardiomyopathy. Created with BioGDP.com.

Epigenetics encompasses several regulatory mechanisms, with methylation being the only known post-translational modification that affects all three central dogma molecules—DNA, RNA, and proteins (15). DNA methylation is the earliest and most extensively studied form (16). Histone methylation plays a crucial role in the activation or repression of genes, cellular differentiation, and developmental processes, and is implicated in various diseases. mRNA methylation, particularly N6-methyladenosine (m6A) modification, is an emerging and rapidly expanding field in epigenetic research. Therefore, this review will focus on these three key methylation modifications (Figure 1). First, we introduce the underlying mechanisms of these three key methylation modifications and explore the impact of metabolic regulation on their kinetics. Next, we will conduct a thorough review and analysis of their pathological mechanisms in the onset of DCM. Finally, we will discuss the clinical translational significance of DNA, histone, and mRNA methylation for diagnosing and treating DCM.

## 2 Classification, overview, and metabolic regulation of methylation

Methylation occupies a central position in epigenetics, serving as a fundamental mechanism for regulating gene expression and cell function, and representing one of the most classical and extensively studied areas in epigenetics. It is widely involved in the processes such as biological development, cellular differentiation, and disease pathogenesis by modifying DNA, histone, or RNA molecules to regulate gene expression patterns and cell fate decisions without altering the DNA sequence. In this section, we specifically focus on how three major methylation mechanisms and metabolic factors regulate epigenetic enzymes and methylation processes. These metabolic influences on methylation may contribute to key pathological processes in DCM, including inflammatory activation, transcriptional reprogramming, mitochondrial dysfunction, oxidative stress, and metabolic remodeling, all of which are further discussed in Section.

### 2.1 DNA methylation

**Figure 2:**
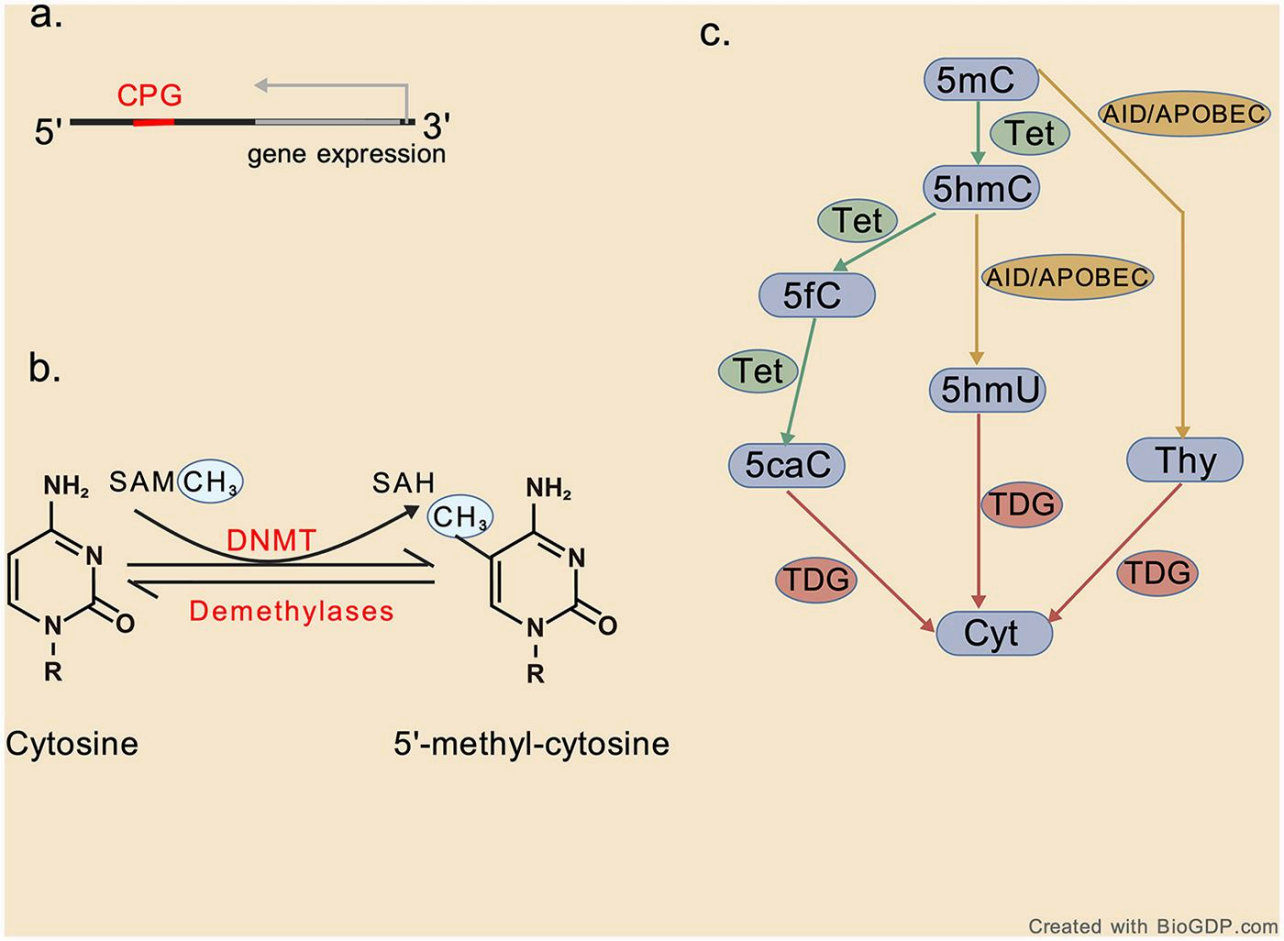
DNA methylation and demethylation. **(a)** The methylation of cytosine primarily occurs on the CpG dinucleotide sequence. **(b)** A chemical modification process in which specific bases on a DNA sequence are catalyzed by DNA methyltransferase (DNMT) to transfer methyl to the fifth carbon atom of cytosine, using s-adenosylmethionine (SAM) as the methyl donor. **(c)** DNA demethylation pathways. The amine group of 5mC can be deaminated (green) by AID/APOBEC, converting 5mC into thymine (Thy). The methyl group of 5mC can be modified by the addition of a hydroxyl group mediated by Tet enzymes to generate 5-hydroxymethyl-cytosine (yellow). AID/APOBEC can deaminate (green) 5hmC to produce 5-hydroxymethyl-uracil (5hmU). In another chemical pathway for 5hmC is that Tet can further oxidize (green) 5hmC to form 5-formyl-cytosine (5fC) and then 5-carboxy-cytosine (5caC). Eventually, the products of each pathway (e.g., Thy, 5hmU, 5fC, and 5caC) are recognized and cleaved off to replace with a naked cytosine mediated by TDG and/or SMUG1, both components of the base excision repair pathway (red). Created with BioGDP.com.

DNA methylation involves the addition of methyl groups to the 5th carbon of cytosine residues within CpG dinucleotides (CpGs), catalyzed by DNA methyltransferases (DNMTs) (Figures 2a,b). This process is frequently associated with gene silencing, a concept first proposed in the 1970s (16). Methylated CpGs are widely distributed across the human genome, particularly in the gene bodies of highly expressed genes, transposable elements, and CpG islands (CGIs)—stretches of DNA enriched in CpG dinucleotides. CGIs are commonly found in gene promoters, and hypermethylation of these regions leads to stable, long-term gene repression by either recruiting transcriptional repressors or blocking transcriptional activators (17). Abnormal methylation of promoter CGIs can disrupt normal heart developmental programs, contributing to congenital heart disease (18). A family of DNMTs catalyzes the transfer of a methyl group (−CH3) from S-adenyl methionine (SAM) to the 5th carbon of cytosine, forming 5-methylcytosine (5mC). DNMT3A and DNMT3B are responsible for *de novo* methylation of unmethylated DNA, while DNMT1 maintains existing methylation patterns during DNA replication, and participates in DNA methylation repair (19). DNA demethylation, on the other hand, can occur passively or actively. Passive demethylation arises in dividing cells due to DNMT1 inhibition or dysfunction. Active demethylation involves chemical modifications of 5mC that are recognized and resolved by the base excision repair (BER) pathway, resulting in the replacement of the modified base with unmodified cytosine. Active demethylation can occur through several mechanisms. One involves the deamination of 5mC to thymine by AID/APOBEC (activation-induced cytidine deaminase/apolipoprotein B mRNA-editing enzyme complex) which creates a G/T mismatch. Another mechanism involves ten–eleven translocation (TET) enzymes, which oxidize 5mC to 5-hydroxymethylcytosine (5hmC) (20). Once 5hmC is formed, it is first oxidized to 5-formyl-cytosine (5fC) and then to 5-carboxy-cytosine (5caC), or deaminated by AID/APOBEC to form 5-hydroxymethyl-uracil (5hmU) (21, 22). Although the physiological role played by 5hmC in regulating DNA demethylation and gene expression has not been fully elucidated, evidence supports 5hmC as an epigenetic mark, not only a demethylation intermediate (23, 24). The mysterious role of 5hmC regulation in modulating DNA demethylation and gene expression warrants further experimental investigation. In all cases, the BER pathway, mediated by thymine DNA glycosylase (TDG), removes the modified base—thymine, 5hmU, 5fC, or 5caC—and replaces it with cytosine (19). TDG is essential for the recruitment of p300 to retinoic acid -regulated promoters, the protection of CpG islands from hypermethylation, and the active demethylation of tissue-specific, developmentally regulated, and hormonally regulated promoters and enhancers (25) (Figure 2c). DNA methylation can be recognized by three distinct families of proteins: *methyl-CpG-binding domain (*MBD) proteins, ubiquitin-like containing plant homeodomain and RING finger domain (UHRF) proteins, and zinc finger proteins. MBD proteins contain conserved MBDs with high affinity for individual methylated CpG sites, with methyl CpG binding protein 2 (MeCP2) being the first identified member of this family (26). The UHRF protein family identifies and binds to methylated cytosine through the SET and RING-related DNA binding domains. Unlike most methyl-binding proteins, which bind to DNA and repress transcription, UHRF proteins function to preserve DNA methylation by interacting with DNMT1 and targeting hemimethylated DNA for maintenance (27). DNA methylation also plays a role in influencing histone methylation. There is evidence that DNA methylation and histone modification have synergistic or interfering effects on protein recruitment for staining. The binding of certain proteins to nucleosomes is regulated by methylation of CpG, H3K4, H3K9, and H3K27 or their combinations. DNA and histone methylation recruit the origin recognition complex (ORC), while DNA methylation interferes with lysine demethylase Fbxl11/KDM2A in identifying methylated nucleosomes on histones (28).

### 2.2 Metabolic regulation of DNA methylation

**Figure 3:**
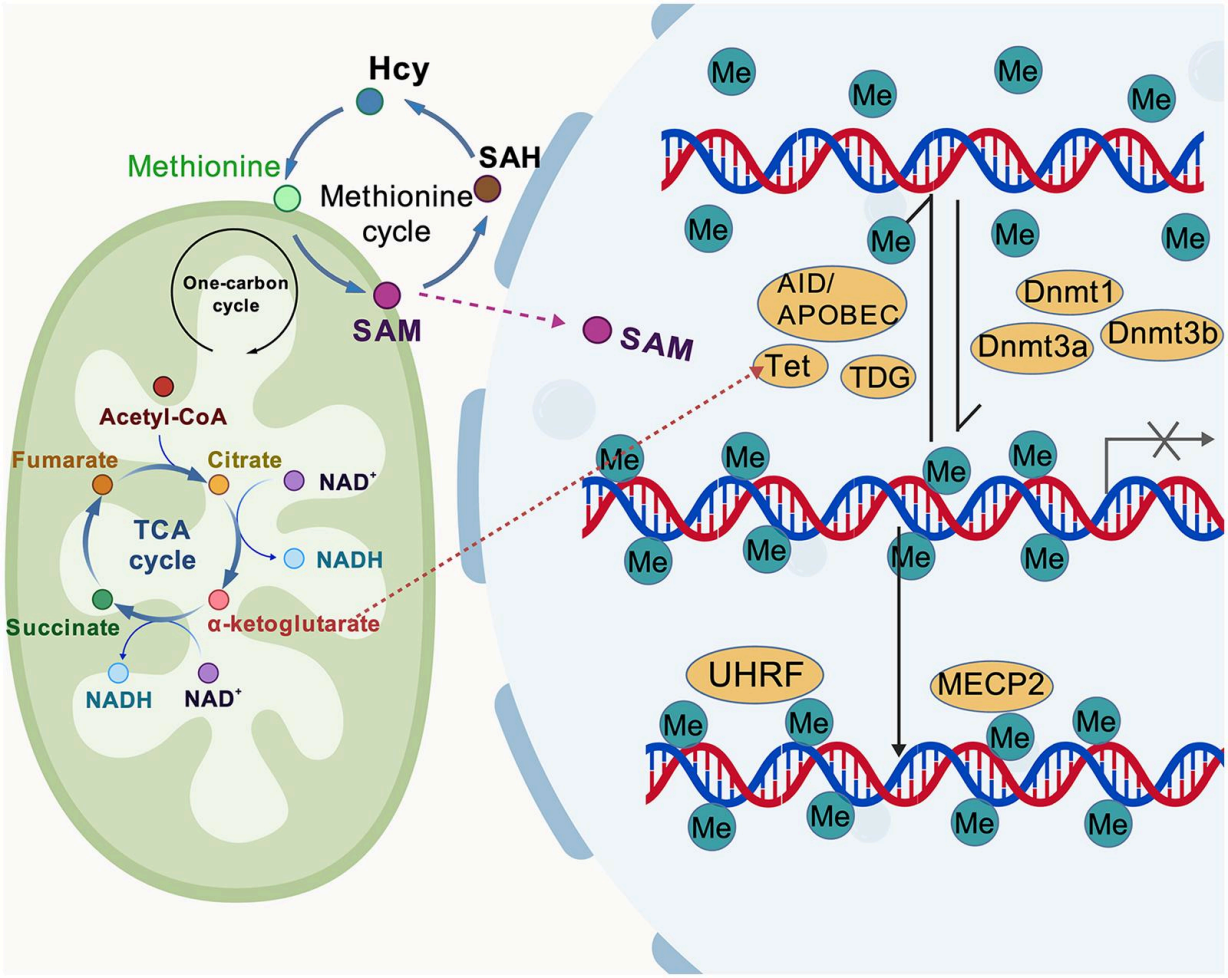
The interplay between metabolism and DNA methylation process. SAM acts as a methyl donor, and the methionine cycle plays a crucial role in regulating methyl donor availability. Accelerating the conversion of homocysteine (Hcy) to methionine can increase SAM, whereas the metabolite S-adenosine-l-homocysteine (SAH) inhibits DNMT activity. TET enzymes require α-ketoglutaric acid (α-KG) as a cofactor. Mitochondrial tricarboxylic acid (TCA) cycle intermediates, including fumarate, succinate, and citrate can affect the content of α-KG. Created with BioGDP.com.

S-adenosylmethionine (SAM) acts as a methyl donor and its increased availability enhances DNMT activity. Strategies to increase the availability of methyl donors include accelerating the conversion of homocysteine to methionine and modulating the uptake of methionine and/or SAM. In contrast, its metabolites—S-adenosyl-l-homocysteine (SAH) and decarboxylated S-adenosylmethionine (dcSAM)—inhibit DNMT activity. DNMT activity is closely related to the concentration of dcSAM and the ratio of dcSAM to SAM. SAH is formed from SAM and serves as an intermediate precursor in the synthesis of homocysteine. Elevated levels of homocysteine are a significant risk factor for the development of various diseases, including cardiovascular diseases. Polyamines, primarily spermine and spermidine, are synthesized from arginine or ornithine. dcSAM provides the aminopropyl group, with ornithine decarboxylase (ODC) acting as the rate-limiting enzyme (29). Spermine counteracts the effects of ODC inhibition, including increased dcSAM levels, reduced DNMT activity, aberrant DNA methylation, and a pro-inflammatory state (30). Nutrition has been shown to mediate epigenetic mechanisms due to its ability to affect the transcriptional activity and expression of specific genes. Diet has an important effect on DNA methylation. For example, high-fat, low-protein, or calorie-restricted diets have been shown to correlate with or alter epigenetic marks (29). Feeding mice an aqueous extract of fruit seeds and peels rich in flavonoids and polyphenols at doses comparable to those given to humans prevents the carcinogen 7,12-dimethylbenz(a)anthracene (DMBA)-induced up-regulation of the *DNMT* gene (31). Ursolic acid, a natural triterpenoid found in blueberries and cranberries, activates the nuclear factor-erythroid factor 2–related factor 2 (Nrf2) pathway and decreases the enzymatic activities of DNMTs (32). TET enzymes require oxygen and α-ketoglutarate (α-KG) as substrates, along with ferrous iron as a cofactor, to mediate demethylation reactions (33). Thus, 2-hydroxyglutarate (2HG), produced by the reduction of α-KG catalyzed by isocitrate dehydrogenase (IDH) enzyme mutants, may impair TET enzyme function and reduce TET2-mediated 5hmC levels (34). This occurs because 2HG occupies the binding site of α-KG within the protein's conformational space (35). In addition, during the tricarboxylic acid cycle, the metabolic products of α-KG—its antagonists—are succinate, fumarate, and itaconate, which all inhibit the activity of TET2 bisoxygenase and reduce demethylation activity (36). Interestingly, not only these metabolites, but also hypoxia decreases the activity of TET2, leading to DNA hypermethylation (37). Vitamin C may enhance TET2 activity, possibly by binding to the catalytic domain of TET proteins, facilitating protein folding, and accelerating oxidation reactions (38). Another agonist of TET is nuclear glutamate dehydrogenase, which interacts with TET3 to provide α-KG to TET3 and increase its demethylation activity in neurons (39) (Figure 3).

### 2.3 Histone methylation

**Figure 4:**
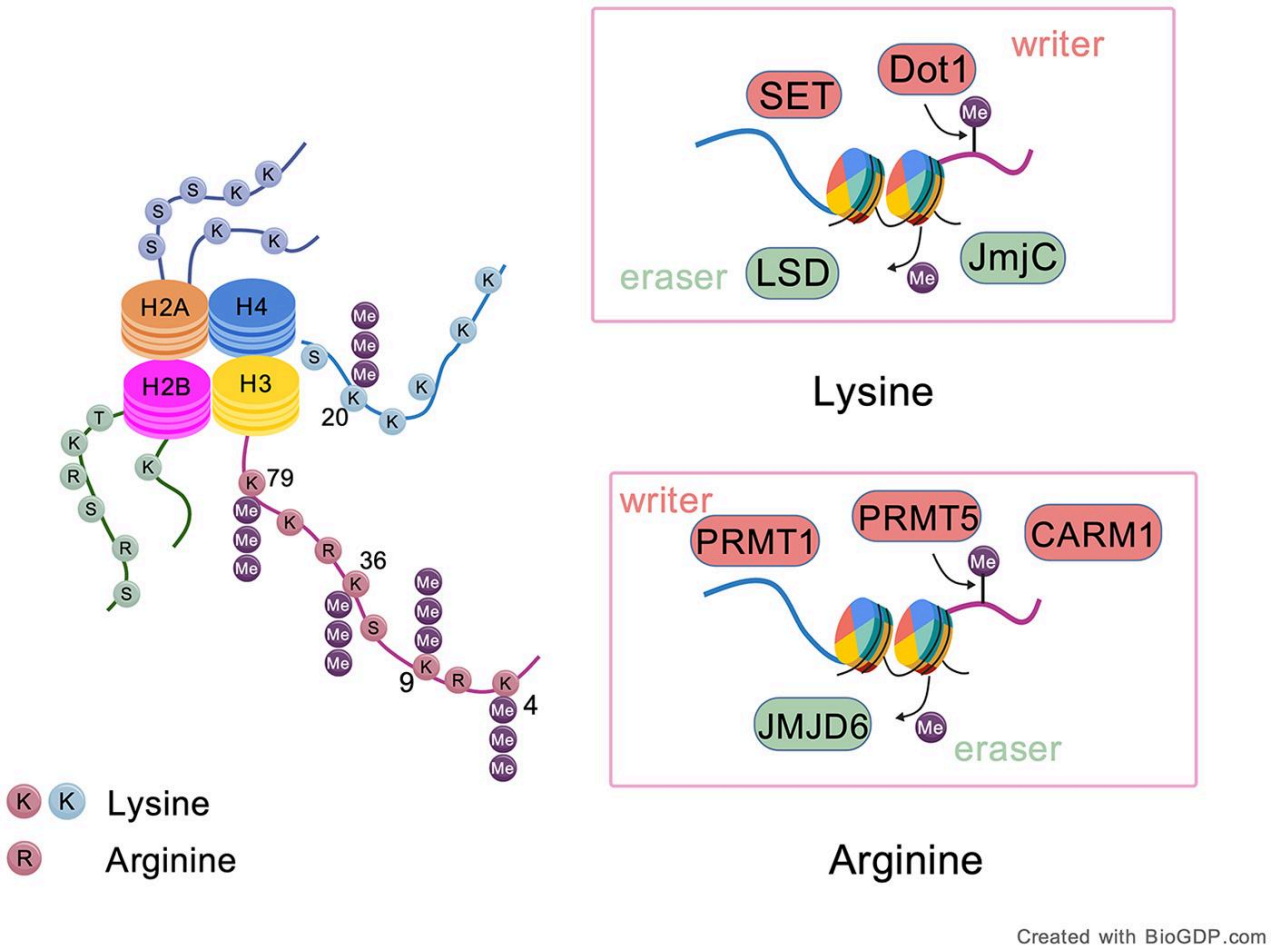
Histone lysine and arginine methylation: sites, enzymes, and functional implications. Key lysine residues include H3K4, H3K9, H3K36, H3K79, and H4K20 among others. Lysine methylation is catalyzed by SET-domain-containing methyltransferases and Dot1-like enzymes, while demethylation is carried out by LSD family and JmjC-domain-containing demethylases. Arginine methylation is installed by PRMTs family or CARM1 and removed by JMJD6. Created with BioGDP.com.

Most of the genetic information in eukaryotic cells is stored in the nucleus as chromatin. The fundamental functional unit of chromatin is the nucleosome, which consists of 147 base pairs of DNA wrapped around a histone octamer made up of two copies each of histones H2A, H2B, H3, and H4 (40). Histone modification includes methylation, acetylation, phosphorylation, adenosine monophosphate, ubiquitination, glycosylation, and more. Histone modification can alter chromatin state by affecting the affinity between histones and the DNA double helix. Gene regulation can also be achieved by influencing the affinity between transcription factors and structural gene promoters (41). Histone methylation was first discovered in the 1960s, catalyzed by histone methyltransferases (HMTs), which transfer methyl groups from SAM to specific target residues of histone lysine or arginine. Currently, HMTs are classified into three families: SET-domain-containing lysine methyltransferases [The denomination of SET structural domain is constituted with 3 initial letters of 3 genes from expressing SET structural domain found at the earliest, which are Su(var)3–9, E(z) and Trx] (42), Dot1-like lysine methyltransferases (KMTs), and protein arginine methyltransferases (PRMTs) (43, 44). One of the most well-established functions of arginine methylation is its role in regulating both constitutive and alternative splicing, primarily mediated by PRMT1/5/7/9 and coactivator associated arginine methyltransferase 1 (CARM1, also PRMT4). Among these, PRMT1 and CARM1 catalyze asymmetric dimethylation at the arginine 3 position of histone H4 (H4R3me2a) and the arginine 17 position of histone H3 (H3R17me2a), respectively—modifications that are commonly associated with active transcriptional promoters. In contrast, PRMT5 can either promote or repress transcription, depending on the specific histone tail residues it modifies (45). The reverse process, histone demethylation, is mediated by histone demethylases (HDMs), which remove methyl groups from lysines or arginines. While arginine demethylases, such as JMJD6, are less well understood (46), lysine demethylases (KDMs) are grouped into two families: amine oxidases (LSDs) utilizes flavin adenine dinucleotide (FAD) as a cofactor and Jumonji C (JmjC)-domain-containing histone demethylases (JHDMs) utilize ferrous iron and α-KG and demethylate tri-methylated lysine (47, 48). Lys residues 4, 9, 27, 36 and 79 of histone H3 (H3K4, H3K9, H3K27, H3K36 and H3K79) and Lys locus 20 of histone H4 (H4K20) are the common and primarily studied loci of methylation. Unlike histone arginine methylation, lysine methylation seems to be a stable mark of gene expression regulation (Figure 4). For example, H3K4, H3K36 and H3K79 methylation is related to gene activation, whereas H3K9, H3K27 and H4K20 methylation are associated with gene silencing (49). There is crosstalk between histone methylation and DNA methylation. *Neurospora crassa* catalyzes the production of H3K9me3 and heterochromatin 1 (HP1), which connects H3K9me3 with DNA methyltransferase DIM-2, with these two methyl markers mutually reinforcing (50).

### 2.4 Metabolic regulation of histone methylation

The changes in methylation status are attributed to differences in enzyme activity between methyltransferases and demethylases. SAM has been confirmed as a universal methyl donor for these enzymes, which transfer methyl groups to substrates, producing SAH and other methylation products. Samantha et al. focused on key histone methylation modifications at the histone tails, which are crucial for maintaining a specific cellular state and have been shown to be metabolically regulated (51). Experimental results revealed that both SAM and SAH were depleted in cells with methionine depletion, and the relative levels of several histone methylation markers decreased, with H3K4me3 showing the most significant change. Additionally, histone methylation responded differently to varying concentrations of methionine, with SAM or SAH concentrations correlating with the kinetic parameters that govern enzyme activity. Therefore, most histone modifications and other methylations may be significantly regulated by methionine metabolism and carbon cycle flux, indicating that SAM and SAH serve as links between one-carbon metabolism and methylation status (52). A recent groundbreaking study found Spic, a member of the ETS family of transcription factors (TFs), binds to enhancer elements and stabilizes NANOG binding to chromatin, particularly at genes involved in choline/one-carbon (1C) metabolism. The 1C metabolic cycle plays a central role in epigenetic regulation by controlling the levels of SAM and SAH. Spic was found to maintains low levels of SAM and decreases the SAM/SAH ratio in 2iL-ESCs, specifically decreasing H3K4me3 levels and increasing H3R17me2a. This study links cellular metabolism to epigenetic regulation in the embryonic stem cells (ESCs) through the function of Spic, and further studies are needed to better understand how SPIC regulates histone methylation in pluripotent cells (53).

Huang et al. found that the TCA cycle enzyme α-ketoglutarate dehydrogenase (KGDH) translocates to the nucleus, where it interacts with various Jumonji C-domain-containing histone demethylases (JMJs) (54). In the nucleus, KGDH catalyzes the oxidative decarboxylation of α-KG, thereby inhibiting α-KG-dependent histone demethylation by JMJs, thus influencing genome-wide gene expression in plants. Notably, the nuclear association of KGDH with JMJs is also observed in mammalian cells, suggesting a conserved mechanism by which KGDH may collaborate with JMJs to regulate histone demethylation and gene expression in mammals (54). The reduction of Fe³⁺ to Fe²⁺ by vitamin C enhances the activity of certain α-ketoglutarate-dependent epigenetic dioxygenases (αKGDDs), including JHDMs. Supplementation with vitamin C leads to a decrease in H3K9me3 and H3K27me3 demethylation, which correlates with the upregulation of osteogenic markers during adipogenic differentiation. The use of L-2-hydroxyglutarate (L-2-HG), a competitive inhibitor of αKGDDs, effectively suppresses the catalytic activity of these enzymes. Remarkably, L-2-HG treatment almost completely blocks the vitamin C-induced reduction in H3K9me3/H3K27me3 levels during osteogenic differentiation of mouse preosteoblasts (23). Such metabolic regulation of histone-modifying enzymes may ultimately affect cardiac remodeling and metabolic adaptation in DCM.

### 2.5 RNA methylation

**Figure 5:**
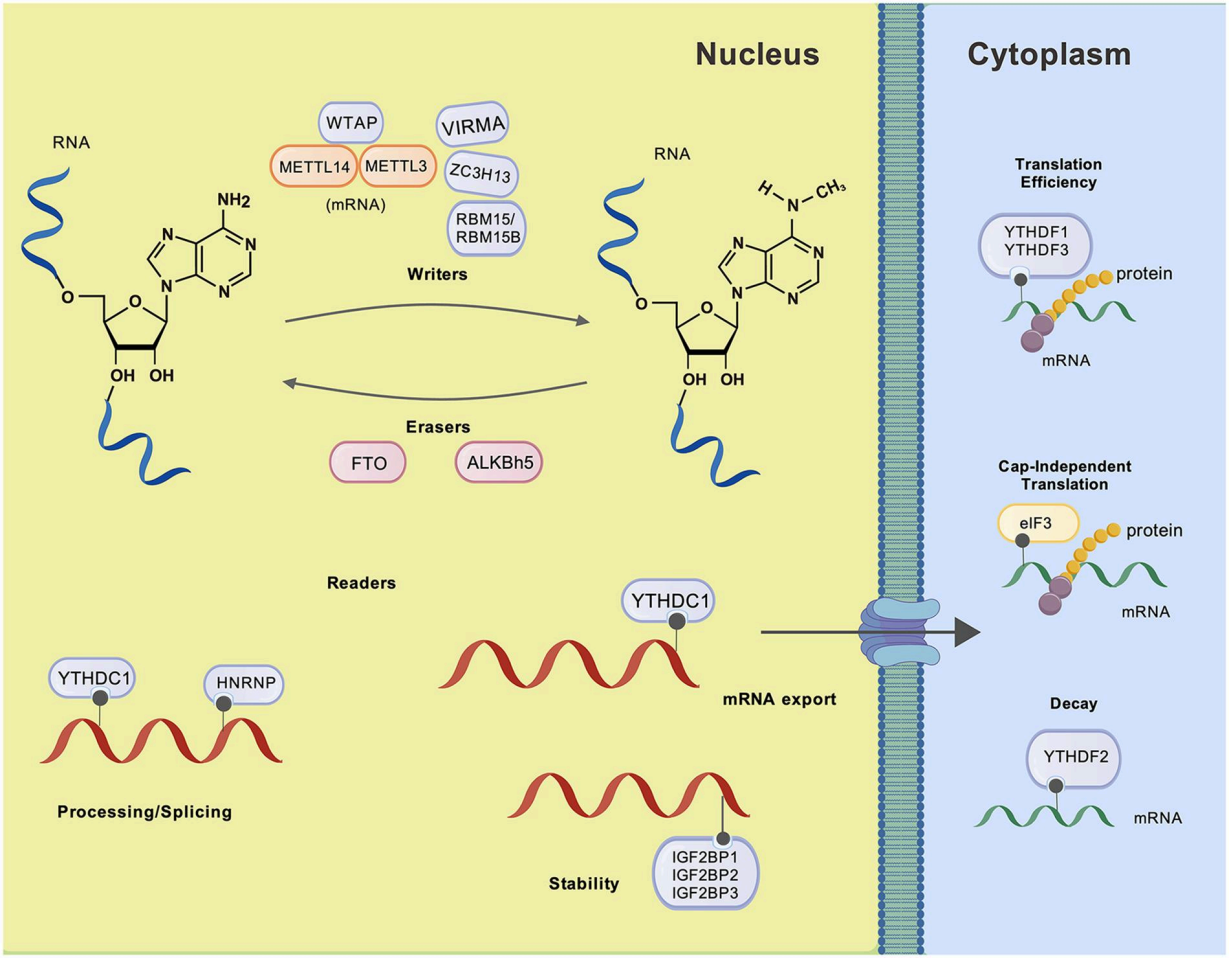
The “lifecycle” of N6-methyladenosine (m6A). The “lifecycle” of m6A methylated mRNA is regulated by m6A “writers” (e.g., METTL3, METTLE14 and WTAP), “erasers” (e.g., ALKBH5 and FTO), and “readers” (e.g., YTHDF1, YTHDF12, YTHDF3, YTHDC1, HNRNP, IGF2BP1/2/3, etc.). Catalyzed by core methyltransferase protein 3 (METTL3), METTLE14 forms a heterodimer with WTAP, which recruits the complexes to specific mRNA targets. ALKBH5 and FTO are responsible for demethylation of m6A-modified RNA. Reader proteins are divided into two categories, one of which is the YTH protein family: YTHDF1 regulates mRNA translation and stability in the cytoplasm; YTHDF2 targets m6A-modified RNA degradation in the cytoplasm; YTHDC1 regulates mRNA splicing and output in the nucleus; YTHDF3 promotes the efficiency of YTHDF1 and YTHDF2. Another category involves heterologous ribonucleoprotein (HNRNP), which primarily regulates the selective splicing or processing of target RNA. Additionally, insulin-like growth factor 2 mRNA binding protein (IGF2BP1/2/3) and eukaryotic initiation factor (eIF) 3 form subfamilies that affect RNA stability and translation. Created with BioGDP.com.

m6A is the most prevalent, reversible, and dynamic RNA modification in eukaryotes, constituting approximately 50% of all methylated ribonucleotides. It is recognized as a crucial post-transcriptional regulatory mark that influences a wide range of RNA types (55). m6A is present in nearly all types of RNA, including mRNA, rRNA, tRNA, snRNA, miRNA, circRNA, and lncRNA, where it plays crucial roles in various pathophysiological processes (56). Recent research highlights its critical role in regulating biological rhythms, cell differentiation, immune function, and other physiological processes (57). Most m6A sites are found in the conserved DRACH motif (D = G/A/U, R = G/A, H = A/U/C) (58), frequently located around stop codons, as revealed by whole-transcriptome m6A mapping. This positioning suggests significant functional roles for m6A modification (59). m6A is installed by methyltransferase complex (called m6A“writers”) including METTL3/14/16 (60, 61), WTAP (62), RBM15, VIRMA, and ZC3H13 (63). Among these, METTL3, the first identified methyltransferase and the core catalytic subunit, plays the major role in m6A deposition. METTL14 interacts with METTL3 to form a stable heterodimer, enhancing the catalytic efficiency of m6A installation on nuclear RNAs. WTAP is a mammalian splicing factor essential for interacting with the METTL3-METTL14 complex. It plays a critical role in the initiation and localization of nuclear speckles, a process necessary for activating m6A methylation. Additionally, WTAP is involved in the recruitment of these complexes to specific mRNA targets (60). On the other hand, demethylases (named m6A“erasers”) such as FTO and ALKBH5 remove m6A modifications, thereby regulating its dynamic nature (64, 65). FTO was the first demethylase identified for m6A and is localized in the nucleus. It is classified as a member of the AlkB-related family of non-heme Fe(II)/α-KG-dependent dioxygenases. FTO exhibits efficient oxidative demethylation activity, specifically targeting m6A residues in RNA. Moreover, FTO is crucial for the biological development of the cardiovascular system (66). The functional effects of m6A are mediated by reader proteins (called m6A“readers”), which recognize m6A-modified RNAs. One class includes YT521-B homology (YTH) domain-containing proteins, which directly bind to m6A-modified transcripts (67). Among these, YTHDF2 targets m6A-modified RNA for degradation at cytoplasmic decay sites. In the nucleus, YTHDC1 regulates mRNA splicing or promotes primary miRNA processing, and promotes nuclear output (68). And when YTHDF3 binds to the translation initiation complex, it promotes the activity of YTHDF1, thereby enhancing the efficiency of YTHDF2 (55). Another category involves heterogeneous nuclear ribonucleoproteins (HNRNPs), which primarily regulate alternative splicing or processing of target RNAs (69). Additionally, insulin-like growth factor 2 mRNA-binding proteins (IGF2BP1/2/3) and eukaryotic initiation factor (eIF) 3 form subfamilies that influence RNA stability and translation (68, 70) (Figure 5). Despite m6A's known significance in cellular function, studies investigating methyltransferases in the context of heart disease remain limited. Expanding research on demethylases and m6A-binding proteins could unveil novel therapeutic targets for cardiac conditions, emphasizing the need for further exploration in this field.

### 2.6 Metabolic regulation of RNA methylation

Metabolic factors are crucial for organisms. Metabolism is typically regulated by RNA transcription and translation, but some metabolites can, in turn, regulate the enzyme activity that controls RNA modification and regulate RNA. Nicotinamide adenine dinucleotide phosphate (NADP) is a reducing agent that participates in many metabolic processes, such as fat synthesis and pentose phosphate pathways. NADP directly binds to FTO and enhances its activity, promoting m6A demethylation and fat formation (71). FTO, like other double oxygenases, also requires vitamin C as a cofactor to achieve full catalytic activity (72). ALKBH5 and FTO m6A demethylase require α-KG, Fe^2+^, and O_2_ to activate their total enzyme activity. Metabolites produced by the TCA cycle regulate m6A demethylation (73, 74). Citrate, a key metabolite in the TCA cycle, can inhibit enzyme activity by binding to the α-KG/FTO complex. More commonly, citrate interacts with the α-KG binding site in ALKBH5, providing a structural basis for understanding the substrate recognition specificity of ALKBH5. This interaction facilitates the exploration of the physiological functions of ALKBH5-mediated m6A demethylation and supports the development of selective therapeutic agents (75). IDHs are key enzyme in the TCA cycle that catalyzes the conversion of isocitrate to α-KG and CO_2_. They also epigenetically control gene expression by affecting α-KG-dependent oxygenase. Enzyme activity in leukemia cells carrying IDH mutations is inhibited, and m6A levels are significantly elevated (76). R-2-hydroxyglutaric acid (R-2HG) is a metabolite produced by mutant IDH that reduces the expression of the FTO gene, inhibits aerobic glycolysis, and diminishes its demethylase activity to increase m6A modification. Interestingly, the inhibition of FTO enzyme activity induced by R-2HG occurs before the downregulation of FTO expression (77).

## 3 Regulation of methylation in dilated cardiomyopathy

### 3.1 DNA methylation in dilated cardiomyopathy

Movassagh et al. demonstrated for the first time that DNA methylation is globally altered in cardiomyopathy, with significant changes particularly enriched in CGIs (78). Haas et al. performed DNA methylation mapping in patients with non-ischemic, idiopathic DCM and identified several aberrant DNA methylation alterations in CGIs: decreased DNA methylation in ERBB3, HOXB13, and ADORA2A, and increased methylation in LY7 (79). These early studies provided pioneering evidence linking DNA methylation to cardiomyopathy and laid an important foundation for subsequent epigenetic investigations in DCM. Meder et al. recently conducted a high-density epigenome-wide DNA methylation analysis, performing the first multi-omics study using left ventricular biopsies and whole peripheral blood from DCM patients and controls. They linked 517 epigenetic loci with DCM and cardiac gene expression, identifying distinct epigenetic methylation patterns conserved across tissues, which supports the potential of using DNA methylation patterns as novel epigenetic biomarkers for DCM diagnosis (80). Jo et al. performed DNA methylation and mRNA expression profiling to identify molecular alterations underlying DCM pathogenesis. Their study identified not only several genes already associated with DCM, such as TBX5 and HAND1, but also novel genes with altered DNA methylation and gene expression patterns (81).

FKBP5 (FKBP prolyl isomerase 5), a protein cochaperone acutely induced by stress, is known to be associated with the inflammatory response in the myocardium, and sterile chronic inflammation is important in the pathogenesis of DCM and heart failure. Recent findings suggest that blood-based epigenetic modifications in FKBP5 may contribute to the pathogenesis of DCM. Researchers found that patients with DCM exhibited significantly lower levels of FKBP5 CpG methylation within the CpG island and promoter region of the genome, along with significantly elevated levels of leukocyte FKBP5 and interleukin-1β (IL-1β) mRNA expression. This was attributed to the suppression of FKBP5 promoter activity under methylated conditions in response to immune stimulation, leading to increased FKBP5 mRNA expression. IL-1β is upregulated via activation of nuclear factor kappa B (NF-κB) in heart failure and has a negative inotropic effect on cardiomyocytes by downregulating Ca2 + reuptake. Additionally, the protein levels of DNMT1 and DNMT3A were significantly reduced in leukocytes from DCM patients (82). Although the data are directly derived from patients with DCM, these findings should be interpreted with caution, as the epigenetic alterations were primarily identified in peripheral blood leukocytes rather than cardiac tissue, which may not fully reflect myocardial-specific regulatory mechanisms.

Genes with changes in 5mC content have been studied in relation to DCM and heart failure, and a study based on the DCM mouse heart model elucidates the role of 5hmC in DCM (78, 79). The authors observed alterations in 5mC and 5hmC levels among several muscle genes in MYBPC3 mutant mouse model of DCM, notably Myh7 (myoglobulin heavy chain 7) and Myh6 (myoglobulin heavy chain 6) which lead to hypertrophy and myocardial phenotype. Low levels of 5mC and 5hmC of Myh6 were observed, indicating epigenetic downregulation in the DCM. GO and KEGG analyses revealed genes enriched in pathophysiological pathways associated with DCM, such as inflammation, tissue fibrosis, cardiac remodeling, and cardiac dysfunction. Interestingly, these pathological changes in DCM mice are more pronounced at 5hmC, indicating a new role in DCM pathogenesis and potentially therapeutic targets (83). In an iPSC-derived cardiomyocyte experiment involving the knockout of the DNMT3A gene, a decrease in the MYH7/MYH6 ratio was observed, leading to a phenotype of reduced contractility. The differential genetic analysis shows that DNA methylation differences are associated with cardiac hypertrophy and increased cardiac proliferation pathways as well as the p53 pathway. Moreover, DNMT3A deficiency led to an upregulation of the lipid metabolism factor PPARγ (peroxisome proliferator-activated receptor gamma), causing mitochondrial swelling and their crest structure damage in knockout cardiomyocytes compared to wild-type cells, indicating a significant reduction in mitochondrial metabolic function (84). However, several important considerations should be taken into account when interpreting these findings. Although DNMT3A knockout is associated with DNA methylation and phenotype changes and downstream alterations in gene expression, it remains difficult to determine whether the observed changes are directly attributable to DNMT3A loss or arise from secondary or compensatory. Indeed, another member of this family, DNMT3B, shares overlapping genomic targets, and therefore, the overall changes in methylation following single-gene deletion are typically limited. While iPSC-derived cardiomyocytes offer improved cellular specificity compared to animal models, they may not fully recapitulate the structural and functional complexity of mature human myocardium.

Mutations in the LMNA gene, which encodes the lamin A/C proteins that interact with the genome through lamin-associated domains (LADs) and regulate gene expression, are a leading cause of DCM and are associated with high mortality in laminopathies. Previous research showed that LADs are redistributed in LMNA-associated DCM, correlating with significant changes in CpG methylation and gene expression (85). Although this study emphasized the role of DNA methylation in DCM, the impact of specific family-specific LMNA mutations was not addressed. A more recent study expanded on this by identifying family-specific four and a half limb domains protein 1 (FHL1) through the comparison of the DNA methylation landscapes in patients from two families with distinct LMNA mutations and control samples. These DMRs revealed inter-family epimutation hotspots near differentially expressed genes, many of which were redistributed in LMNA-related DCM. Fibroblast DMRs were found to be enriched in distal regulatory features, transcriptionally repressed chromatin, and genes associated with phenotypic traits seen in tissues affected by laminopathies. Cardiac disease such as DCM remains the most common type of LMNA-associated disease called laminopathy. Notably, epistatic mutations in DCM appear to arise primarily in a family-specific manner (86). As current evidence is derived from a limited number of families and may not fully capture the broader heterogeneity of LMNA-associated DCM. In addition, differences in genetic background and sample types may contribute to variability in observed methylation patterns, highlighting the need for larger, well-characterized human cohorts to validate these observations.

Taken together, current evidence highlights DNA methylation as an important regulatory layer in DCM, with early pioneering studies establishing global methylation alterations and subsequent multi-omics analyses linking these changes to gene expression and potential biomarker development. Mechanistic studies further suggest that DNA methylation may influence key pathological processes, including inflammation, metabolic remodeling, and contractile dysfunction. However, the field remains limited by substantial heterogeneity in experimental models, differences between peripheral and myocardial tissues, and challenges in distinguishing causal effects from secondary or compensatory changes. In particular, findings derived from animal models, *in vitro* systems, or small patient cohorts may not fully capture the complexity and variability of human DCM.

### 3.2 Histone methylation in dilated cardiomyopathy

Pathological remodeling can lead to cardiac enlargement, fibrosis, conductivity abnormalities, and heart failure. A hallmark of it is the reactivation of fetal genes that were suppressed during development. This transcriptional reprogramming can occur through epigenetic modifications. In a cardiac-specific JMJD2A/ KDM4A knockout mouse model, the response to transverse aortic constriction (TAC)-induced pathological cardiac hypertrophy was attenuated. In contrast, overexpression of Jmjd2a significantly increased the expression of fetal cardiac gene markers (such as ANP, BNP, and Myh7), suggesting that JMJD2A promotes cardiac hypertrophy under pathological conditions. This effect is mediated by JMJD2A through targeting FHL1 and increasing H3K9 trimethylation (87). The TAC model primarily represents pressure overload–induced cardiac hypertrophy and subsequent heart failure; however, these pathological changes cannot be directly equated with DCM, despite shared features such as fetal gene reactivation, fibrosis, and ventricular remodeling. The pro-hypertrophic effect of JMJD2A observed in the TAC model may therefore reflect pressure overload–specific adaptive or maladaptive responses, rather than mechanisms that directly drive dilated cardiomyopathy. H3K9me2 dimethyltransferase EHMT1/2 can prevent left ventricular hypertrophy (LVH) by increasing overall H3K9me2 levels, and experimental results indicate that preventing H3K9 methylation loss by targeting miR-217 and EHMT1/2 is a potential therapeutic strategy (88). In cardiomyocytes, KDM3A can activate the Timp1 transcription with fibrosis-promoting activity, thereby promoting LVH and fibrosis in stress-overloaded models, while the KDM inhibitor JIB-04 can inhibit disease progression and mitigate this pathological remodeling (89). Genes that affect oxidative metabolism also undergo transcriptional reprogramming. Prior to DCM onset in mice, the absence of lysine demethylase 8 (Kdm8) in myocardial cells increases H3K36me2, activates Tbx15, and thereby inhibits NAD + pathways leading to hypoxia in myocardial cells. T-box factor (Tbx) is a family of transcription activators and repressors, while Tbx15 is a metabolic master regulator associated with reduced mitochondrial mass, decreased respiratory function, and fat cell browning. NAD + treatment is sufficient to inhibit adverse myocardial remodeling and DCM progression in mice with Kdm8 mutations (90). Taken together, these studies highlight the critical role of histone methylation dynamics in regulating transcriptional reprogramming associated with pathological cardiac remodeling. These epigenetic modifications contribute to the shift in cardiomyocyte phenotype, linking structural remodeling with metabolic dysfunction.

Studies have shown that implantation of a left ventricular assist device (LVAD) often reverses left ventricular (LV) remodeling in patients with end-stage nonischemic DCM. One study found that H3K4me3, H3K9me2, H3K9me3, and H4K20me3 were significantly underexpressed in the LV prior to LVAD implantation. Notably, the expression of these four histone methylation-related molecules significantly increased following LVAD support, partly due to upregulation of H3K9 methyltransferase and SUV39H1, alongside downregulation of H3K9 demethylase and JMJD in the LVAD-supported LV. Furthermore, the expression levels of atrial natriuretic peptide (ANP) and brain natriuretic peptide (BNP) were negatively correlated with those of H3K4me3, H3K9me2, and H3K9me3 (91). The dynamic restoration of histone methylation marks following LVAD support highlights the potential of epigenetic modifications as responsive and potentially modifiable regulators in advanced heart failure. In patients with heart failure, the reactivation of fetal gene programs, including ANP and BNP, serves as a marker of maladaptive remodeling in the LV. During the early stages of increased hemodynamic load, histone deacetylase 4 (HDAC4) translocates to the nucleus, leading to the demethylation of H3K9, dissociation of HP1 from promoter regions, and activation of the ANP gene. Surprisingly, no significant changes in histone acetylation were observed in the promoter regions of these genes. This may be due to HDAC4's association with the histone methyltransferase SUV39H1, facilitating its recruitment to the promoter regions of ANP and BNP, thereby increasing H3K9 methylation. This recruitment diminishes with the nuclear export of HDAC4 in response to elevated cardiac load (92).

Jumonji domain protein D3 (JMJD3, KDM6B) is a member of the histone demethylase family and specifically catalyzes the demethylation of H3K27me3 to H3K27me2/1, a process essential for normal organ development. JMJD3 is upregulated in myocardial hypertrophy induced by isoproterenol, which may promote the expression of β-myoglobin heavy chain (β-MHC) through demethylation of H3K27, leading to myocardial hypertrophy (93). Their recent study further investigated the effects of JMJD3 on doxorubicin (DOX)-induced cardiomyopathy and its potential mechanisms. The Sestrins (SESNs) protein family is a class of stress-induced metabolic proteins that can reduce reactive oxygen species (ROS) production. In both *in vivo* and *in vitro* DCM models induced by doxorubicin, JMJD3 expression is increased while SESN2 expression is reduced. Knocking down JMJD3 expression or inhibiting JMJD3 function with GSK-J4 can effectively alleviate DOX-induced chronic cardiopathy and mitochondrial dysfunction. Mechanically, JMJD3 inhibits the expression of mRNA and proteins by reducing H3K27me3 enrichment in the SESN2 promoter region, thereby inducing apoptosis and mitochondrial damage in myocardial cells (94). Although the JMJD3–SESN2 axis provides a plausible mechanistic link between histone methylation and oxidative stress regulation, given that DOX itself strongly induces ROS generation and mitochondrial damage, it remains unclear whether the observed changes in mitochondrial function and apoptosis are directly mediated by JMJD3-dependent chromatin remodeling or instead reflect broader stress responses induced by DOX exposure. Moreover, distinct downstream effects have been observed under different metabolic contexts (e.g., isoproterenol vs. doxorubicin exposure), highlighting the need for more specific models to investigate its role in DCM.

The Lys 79 methylation of histone H3 is mediated by DOT1 and its mammalian homolog, DOT1L. Nguyen et al. conducted mechanism studies and found that DOT1L regulates the stability of the dystrophin-glycoprotein complex, which is crucial for myocardial cell viability, by regulating the transcription of dystrophin (Dmd). Cardiac-specific knockout of DOT1L leads to ventricular dilation, increased cardiomyocyte death, impaired contractile function, and conduction abnormalities. While, expression of miniDmd can rescue these DCM-like phenotypes, highlighting its role as an important target in the impact of DOT on myocardial cells (95). H3K4-specific HMT MLL3 containing SET domains is significantly elevated in the left ventricle samples of DCM patients, with its substrate H3K4 methylation significantly increased indicating gene activation. The levels of downstream regulated proteins, including Smad3, GATA4, and EGR1, were significantly increased through activation by MLL3-mediated H3K4 methylation. The cardiac model induced by TAC shares many pathological changes with DCM hearts, such as myocardial cell hypertrophy and cardiac fibrosis. In the hearts of mice that received TAC, the mRNA levels of MLL3 were similarly significantly elevated and were closely related to the diameter at the end of left ventricular diastole and the left ventricular ejection fraction (96). This study provides relatively comprehensive evidence by integrating human DCM myocardial samples with findings from a TAC-induced mouse model. However, SETD1A mRNA was unexpectedly decreased in mouse hearts with 8 weeks of TAC. The authors proposed that this discrepancy in expression patterns may primarily arise from the inability of TAC-induced cardiac remodeling to fully recapitulate the pathological processes of DCM, which is consistent with the limitations of the TAC model discussed above. Chen et al. concluded using a rat DCM model induced by furazolidone that H3K9 histone methyltransferase G9a reduces DCM risk by regulating cell adhesion molecules (CAMs). CAM is highly expressed in diseased human hearts and may play a significant role in the mechanisms of heart failure by mediating immune responses, making it a cause of chronic heart failure. G9a reduces its levels by silencing the CAM gene (97).

Taken together, these studies underscore the critical role of histone methylation in regulating transcriptional reprogramming during pathological cardiac remodeling, linking fetal gene reactivation, fibrosis, and metabolic dysfunction to changes in chromatin state. However, the models used in these studies differ substantially in etiology and may not fully recapitulate DCM. It remains challenging to distinguish direct epigenetic regulation from secondary responses driven by oxidative stress, metabolic perturbations, or cardiac injury. Furthermore, the dual role of histone methylation in gene activation and repression, together with the context-dependent functions of its key enzymes under different stress conditions, highlights the complexity of histone methylation–mediated regulation.

### 3.3 m6A in dilated cardiomyopathy

Reversible mRNA modifications have been proposed in 2010 by the He laboratory (98). Kmietczyk et al. were the first to investigate and elucidate the critical role of mRNA epi-modification, specifically m6A, in regulating gene expression in the heart. By measuring m6A levels and sequencing m6A-specific immunoprecipitation mRNA, it was found that m6A levels were elevated in the DCM myocardium, along with increased levels of METTL3 protein and RNA in a small portion of DCM hearts. Knockdown of METTL3 significantly increases the expression of hypertrophic markers *Nppa* and *Nppb*, leading to increased cardiomyocyte size; FTO knocking down inhibits hypertrophy. In mouse models induced by TCA surgery for pathological hypertrophy, the overexpression group of METTL3 showed a reduction in fibrosis (99). This study demonstrates that mRNA modification is a way to control myocardial gene expression, laying the groundwork for mechanisms and experimental methods to investigate the impact of mRNA methylation on DCM.

Cardiomyocyte death is at the core of pathological development in various cardiomyopathy conditions, with m6A profoundly influencing the different forms of cell death involved (100). Ferroptosis is a type of iron-dependent regulatory cell death characterized by lipid peroxidation and plays a crucial role in cardiotoxicity induced by DOX. Overexpression of FTO can significantly improve cardiac function and myocardial cell viability following DOX treatment, and it is known that P21 inhibits ferroptosis through interaction with Nrf2. Mechanically, FTO upregulates P21/Nrf2 by mediating P53's m6A demethylation or directly by mediating P21/Nrf2's m6A demethylation, thereby inhibiting ferroptosis induced by DOX. This process is regulated by the human antigen R (HuR) (101). The mechanism by which METTL3 mediates ferroptosis in cardiomyocytes is still unclear, but a new study shows that knocking down METTL3 can inhibit the m6A methylation of the cystine/glutamate antiporter SLC7A11. Moreover, silencing METTL3 can inhibit the reader YTHDF2 from recognizing the m6A methylation of SLC7A11, thereby suppressing ferroptosis (102). METTL3 also affects autophagy, which helps clear damaged mitochondria and proteins in DCM; however, excessive and insufficient autophagy can also harm myocardial health. METTL3 inhibits autophagy by adding m6A modifications to the 3'-UTR of the transcription factor EB mRNA. TFEB binds to the promoter of ALKBH5 and activates its transcription, whereas the suppression of METTL3 is achieved by reducing mRNA stability, thereby forming a negative feedback loop (103). However, the causal directionality and stability of this feedback remain insufficiently validated. The coexistence of METTL3-mediated repression of TFEB and compensatory TFEB upregulation upon METTL3 knockdown suggests a complex regulatory balance rather than a linear pathway. Moreover, given that TFEB is a central regulator of lysosomal biogenesis and autophagy, its activation may override upstream epigenetic constraints under stress conditions. Therefore, whether the METTL3–TFEB interaction represents a stable regulatory circuit or a transient stress response remains to be further elucidated. Various forms of cell death, including apoptosis, necroptosis, ferroptosis, and autophagy is a focal point of research into various disease mechanisms. Epigenetics is closely related to the mechanisms of cell death in the development of dilated cardiomyopathy and should be subject to more in-depth study to identify therapeutic targets.

Recent evidence links the onset of DCM to disruptions in the immune microenvironment, abnormal infiltration of immune cells in patients’ myocardial tissue, and overexpression of pro-inflammatory cytokines. Single-sample gene set enrichment analysis showed a high infiltration abundance of CD8^+^ T lymphocytes, NK cells, monocytes, and B lymphocytes in DCM myocardial tissue. Single-cell RNA-Seq identified that mechanically, IGFBP2-mediated m6A enhances immune cell infiltration, activates checkpoint, MHC-I, and T cell co-stimulation signaling pathways, thereby increasing the risk of DCM. This makes IGFBP2 a potential therapeutic target for DCM (104). By analyzing the differences and correlations in the GEO-Merge database, it was shown that the DLAT and LDHA genes affect m6A methylation in DCM and exhibit varying degrees of correlation with activated dendritic cells, activated mast cells, and other immune cells (105). However, database analysis alone cannot prove that DLAT and LDHA regulate the causal relationship between m6A and immune cell subpopulations and DCM; further experiments are needed to verify this. METTL3 promotes maturation of *pri-miR-193a* by enhancing m6A modifications, leading to downregulation of BCL2 like 2 (BCL2L2) and thereby activating the apoptosis pathway. This harmful axis plays a crucial role in apoptosis and inflammatory responses in cardiomyopathy cells (106). Some pro-inflammatory cytokines in DCM myocardium activate the transforming growth factor β/SMAD signaling pathway, leading to fibroblast activation and excessive extracellular matrix deposition, which exacerbates myocardial fibrosis and ultimately results in ventricular remodeling and severe cardiac contraction and relaxation (63). Studies on MI mouse models have shown that overexpression of FTO can improve cardiac function by increasing ejection fraction and enhancing ventricular wall movement. Further investigation into the underlying mechanisms revealed that FTO overexpression can reduce fibrosis and enhance angiogenesis (107). METTL3 promotes collagen deposition, fibroblast proliferation, and activation by directly interacting with downstream target long non-coding RNA MetBil to enhance cardiac fibrosis and regulate the m6A methylation of fibrosis-related genes (108). Notably, METTL3 exerts divergent effects across multiple pathological dimensions. On the one hand, METTL3 knockdown promotes hypertrophic remodeling and fibrotic responses, while METTL3 overexpression appears to attenuate fibrosis in stress-induced models, suggesting a protective role in structural remodeling. On the other hand, suppression of METTL3 inhibits ferroptosis through stabilization of SLC7A11 and reduced YTHDF2-mediated mRNA degradation, thereby preserving cardiomyocyte viability. In addition, METTL3-dependent pathways have been implicated in promoting apoptosis and inflammatory responses via miRNA-mediated mechanisms. Consequently, therapeutic targeting of METTL3 requires careful consideration, as its inhibition or activation may yield beneficial effects in one pathological process while exacerbating others. Further studies like multi-omics approaches are necessary to resolve these opposing roles and to determine whether selective modulation of METTL3-dependent pathways can achieve net clinical benefit in DCM.

## 4 Translational perspectives

DCM lacks targeted therapies, and most patients are managed with β-blockers, angiotensin-converting enzyme inhibitors, mineralocorticoid receptor antagonists, and SGLT2 inhibitors to mitigate disease progression. Despite these interventions, the long-term prognosis remains unsatisfactory. Furthermore, the underlying mechanisms driving DCM onset are not yet fully understood, and there is a significant lack of reliable clinical biomarkers for its diagnosis, prevention, and monitoring (105). Several novel risk stratification tools have been developed to enhance prognosis stratification for heart failure and sudden cardiac death, and to develop drugs targeting and correcting potential molecular or genetic defects. For example, LMNA variant cardiomyopathy has a higher risk of progressing to refractory heart failure due to other factors, thus, the LMNA-specific ventricular tachyarrhythmia calculator is used for LMNA variant cardiomyopathy patients to choose treatment options and assess prognosis (109). As previously discussed that LADs influence the methylation pattern at CpG sites to induce LMNA mutations-related DCMs, manifesting as high methylation and gene inhibition. Path analysis identified p53 (or TP53) as the primary activation pathway (85). Therefore, studying the differential expression of LADs in response to CpG methylation and lncRNAs may provide solutions for DCM treatment regimens. Furthermore, how p53 affects gene expression in the DCM heart, and which biological functions or processes lead to DCM require more in-depth study. These differentially expressed genes are likely to become targets for treating DCM.

Decitabine, also known as 5-aza-dC, is a DNA methyltransferase inhibitor that is known to improve atherosclerosis. This is achieved by reducing the DNA methylation state of liver X receptor α and PPAR*γ*1, thereby inhibiting these transcription factors which regulate macrophage inflammation in the development of atherosclerosis (10). The finding suggests that DNMT inhibitors such as decitabine may have potential therapeutic relevance in DCM by modulating macrophage-mediated inflammation and subsequent fibrotic remodeling. Given that macrophages and other immune cells directly activating fibroblasts and indirectly producing fibroblasts that promote fibrosis is a typical pathological feature of DCM, targeting DNA methylation in immune cells may represent a plausible strategy to attenuate disease development. However, it should be noted that the current evidence is primarily derived from studies in atherosclerosis and other inflammatory conditions rather than DCM-specific models. Therefore, whether similar epigenetic mechanisms operate in the context of DCM remains to be fully established. 5-aza-dC also has an impact on heart failure. Heart failure is typically associated with enhanced sympathetic nervous system activity and elevated catecholamine levels, with norepinephrine-mediated whole-genome DNA methylation showing concentration-dependent increases, leading to left ventricular hypertrophy and failure. And 5-aza-dC treatment can eliminate promoter hypermethylation, save protein kinase C epsilon (PKCɛ) gene expression, and reverse such phenotypes. Moreover, it can also protect the heart from ischemic and perfusion damage (110). Pathological cardiac remodeling serves as a frequent connecting pathway between primary dilated cardiomyopathy and the development of heart failure. 5-azapine (5-aza) can bind to and inhibit DNMT, and this study shows that it exhibits anti-fibrotic and anti-fatigue effects by reducing the expression of relevant genes. In ventricular fibroblasts treated with 5-aza, levels of type I collagen, type III collagen, and α-smooth actin were all reduced. Heart *MYBP-C3* is a gene associated with cardiac hypertrophy, and inhibiting DNA methylation may improve cardiac function by upregulating this gene (111). Non-nucleoside DNMT inhibitor RG108 can also alter gene expression to weaken the activation of related genes. Studies show that hypertrophy and fibrosis pathological changes in TAC-induced rat models were reduced and contractility improved after RG108 treatment (112).

H3K9 methyltransferase inhibitor chaetocin can improve myocardial hypertrophy and treat heart failure. This is because it reverses the excessive heterochromatinization of important cardiac pump-related genes caused by H3K9me (113). H3K27 methyltransferase (EZH2) regulates extracellular matrix remodeling and fibroblast activation, and additionally influences the inflammatory microenvironment through inflammation-related transcriptional networks, such as NF-κB pathways and inflammatory cytokine expression, promoting the transition from chronic inflammation to fibrosis. Wei et al. expanded the role of EZH2 from a cancer epigenetic target (e.g., clinically approved tazemetostat) to a potential therapeutic target in non-cancerous chronic inflammation and fibrosis (114). Although direct evidence in dilated cardiomyopathy is still lacking, these findings suggest that targeting EZH2-mediated histone methylation may represent a potential therapeutic strategy for cardiac remodeling and heart failure. Given that the therapeutic effects of HDAC inhibitors in cardiovascular diseases have been relatively well established, we also mention them here, as future crosstalk mechanisms may potentially inform methylation-targeted therapies. For instance, resveratrol reduces endothelial nitric oxide synthase (eNOS) for treating atherosclerosis and coronary heart disease (115); selective Class I HDAC inhibitor MS-275 (entinostat) increases catalase expression, reduces myocardial infarction area, and improves cardiac systolic function after ischemia–reperfusion (116). In summary, although targeting methylation-related pathways offers promising avenues for modulating key processes such as inflammation, fibrosis, and cardiac remodeling, current evidence is largely derived from non-DCM models and remains predominantly preclinical. The immediate translational application is limited by the lack of DCM-specific studies and clinical trials, as well as concerns regarding specificity, off-target effects, and systemic toxicity and side effects. Nonetheless, these findings provide a conceptual framework for future investigation, and further DCM-focused studies may facilitate the development of more precise epigenetic therapies.

## 5 Discussion

Previous reviews have comprehensively elaborated on DNA methylation, histone modification, non-coding RNA, chromatin remodeling, and all the modifications they contain. This review instead focuses on three forms of methylation epigenetic regulation involving DNA, histones, and mRNA, offering a novel perspective. First, rather than functioning as independent regulatory layers, DNA methylation, histone methylation, and m6A RNA modification may form a coordinated, multi-level regulatory network in DCM. At the transcriptional level, DNA methylation and histone methylation jointly shape chromatin accessibility and gene expression programs, thereby influencing key processes such as fibrosis, hypertrophy, and metabolic remodeling. At the post-transcriptional level, m6A modification further fine-tunes gene expression by regulating mRNA stability, translation efficiency, and stress responses, particularly in pathways related to cardiomyocyte survival and cell death. Next, all three forms of methylation are closely interconnected through common metabolic substrates and cofactors. For instance, S-adenosylmethionine (SAM) serves as the universal methyl donor for DNA, histone, and RNA methylation reactions, while metabolites such as S-adenosylhomocysteine (SAH), α-ketoglutarate, and NAD + critically influence the activity of DNA demethylases, histone demethylases, and RNA-modifying enzymes. Importantly, many of these metabolites are directly linked to the tricarboxylic acid (TCA) cycle. For example, α-KG is a key intermediate of the TCA cycle and an essential cofactor for dioxygenases such as TET enzymes and JmjC-domain-containing histone demethylases, whereas alterations in mitochondrial metabolism can affect NAD + availability and cellular redox balance. Therefore, perturbations in TCA cycle activity—such as those observed in DCM, including mitochondrial dysfunction, impaired oxidative phosphorylation, and substrate utilization shifts—are closely associated with metabolic disturbances such as impaired oxidative phosphorylation and altered substrate utilization. In this context, metabolic remodeling in DCM may not only represent a downstream consequence of cardiac dysfunction but also act as an upstream regulator that reshapes the epigenetic landscape and contributes to disease progression. Finally, there is crosstalk among the three mechanisms, such as synergistic or antagonistic effects between DNA methylation and histone methylation. As previously mentioned, DNA methylation and histone marks such as H3K9me3 can act synergistically to recruit chromatin-associated complexes and stabilize transcriptionally repressive heterochromatin. Conversely, DNA methylation can also interfere with the recognition of histone marks by specific demethylases, thereby modulating chromatin accessibility and transcriptional outcomes. Emerging evidence also supports the existence of bidirectional crosstalk between m6A RNA modification and histone methylation. On the one hand, histone marks such as H3K36me3 can facilitate the recruitment of the m6A methyltransferase complex to actively transcribed regions, thereby promoting co-transcriptional m6A deposition. On the other hand, m6A modification can influence chromatin states through reader proteins (117). However, studies directly addressing m6A–histone crosstalk remain scarce. Despite increasing recognition of individual epigenetic mechanisms, studies investigating crosstalk among DNA methylation, histone methylation, and m6A RNA modification remain markedly limited. Given their shared dependence on metabolic substrates and their convergent roles in regulating key pathological processes such as fibrosis, hypertrophy, and cardiomyocyte survival, it is plausible that these epigenetic mechanisms function in a coordinated and interdependent manner. Elucidating the interactions among these epigenetic layers may provide deeper mechanistic insight into DCM pathogenesis and open new avenues for combinatorial or multi-target epigenetic therapies.

## 6 Limitations

While this review provides an overview of three major forms of methylation regulation, the impact of metabolic factors on methylation-related enzymes, and the underlying methylation mechanisms in DCM, several important limitations should be acknowledged. First, a major challenge lies in the heterogeneity of disease models. Many studies included in this review are derived from diverse experimental systems, including genetic DCM, pressure overload models, doxorubicin-induced cardiomyopathy, and general heart failure. Although these models share certain pathological features, such as fibrosis and ventricular remodeling, their underlying mechanisms differ substantially, which may limit the applicability of the findings to other forms of DCM. Second, differences between human myocardial tissue, animal models, and *in vitro* systems are not always systematically addressed, and inconsistencies across studies are often difficult to reconcile. Third, although this review attempts to integrate DNA methylation, histone methylation, and m6A RNA modification, studies investigating the crosstalk among these layers remain limited. In addition, the integration of metabolic remodeling with epigenetic regulation remains incomplete. direct evidence from human myocardial metabolomic studies in DCM is still limited. Finally, the translational potential of targeting methylation pathways remains constrained. Most available medicines are not DCM-specific, but are instead applied in the treatment of atherosclerosis, pressure overload, or general heart failure. While these conditions share overlapping features such as inflammation, fibrosis, and pathological remodeling, their underlying etiologies differ, making it uncertain whether these findings can be directly extrapolated to DCM. Despite encouraging preclinical results, methylation-targeting agents, including DNMT inhibitors and histone methyltransferase inhibitors, have not yet entered clinical trials for DCM, highlighting a significant gap between experimental research and clinical application.

Future studies should focus on DCM-specific, multi-omics approaches integrating epigenetic, transcriptomic, and metabolic data, as well as the development of cell-type–targeted and context-specific epigenetic therapies. Such efforts will be essential to translate epigenetic insights into clinically relevant interventions for DCM.
